# Supplementary material for: Cyber-victimization and its association with depression among Vietnamese adolescents
Source: PeerJ. 2022 Feb 9;10:e12907. doi: 10.7717/peerj.12907 (PMC8840053; doi:10.7717/peerj.12907)
Supplement: Supplemental Information 2 [file peerj-10-12907-s002.pdf]

English version

The following questions ask about your experiences of school connectedness, family life, society, mental health, and cyberbullying. This survey is not a test. There are no right or wrong answers. PLEASE DO NOT TELL ANYONE ELSE ABOUT YOUR ANSWERS. WE WILL KEEP YOUR ANSWERS CONFIDENTIAL, AND NO ONE WILL KNOW WHO YOU ARE THROUGH YOUR ANSWERS. Please read each statement, decide your answer, then respond by selecting the one that best matches.

BACKGROUND INFORMATION

Your gender	☐ Male ☐ Female
Which grade are you in?	☐ Grade 8 ☐ Grade 9 ☐ Grade 10 ☐ Grade 11 ☐ Grade 12
What has been your grade point average in the last semester?	☐ Very below average ☐ Below average ☐ Average ☐ Fairly good ☐ Good
How often do you access the Internet?	☐ All of the time ☐ A little of the time<br>☐ Several times/week ☐ None of the time<br>☐ Several times/month
On average, how many hours per day do you spend on accessing the Internet?	☐ <2 hours/day ☐ 2-4 hours/day ☐ >4 hours/day
Where do you usually access the Internet? **(You can select multiple answers)**	☐ At home in a private room<br>☐ At home in the living room when no one<br>☐ At home in the living room when relatives<br>☐ Public places (coffee shops)<br>☐ At school during school hours<br>☐ At school outside of school hours<br>☐ Other: ……………………………
Which types of devices do you use to connect to the Internet? **(You can select multiple answers)**	☐ Mobile phone<br>☐ Tablet<br>☐ Shared computer<br>☐ Personal computer<br>☐ Other: ……………………………
What do you usually do on Internet? **(You can select multiple answers)**	☐ Accessing social networking sites (Facebook, Youtube,...)<br>☐ Chatting with others<br>☐ Sending an email<br>☐ Searching for information<br>☐ Reading the news<br>☐ Studying<br>☐ Listening to music<br>☐ Watching movies<br>☐ Shopping<br>☐ Playing games<br>☐ Posting photos<br>☐ Other: ……………………………
Currently, who are you living with? **(You can select multiple answers)**	☐ Both father and mother<br>☐ Father or mother<br>☐ Relatives<br>☐ Alone<br>☐ Other: ……………………………

INTERNET ADDICTION TEST

	Rarely	Occasionally	Frequently	Often	Always
How often do you find that you stay on-line longer than you intended?	☐	☐	☐	☐	☐

How often do you neglect household chores to spend more time on-line?	☐	☐	☐	☐	☐
How often do you prefer the excitement of the Internet to intimacy with your partner?	☐	☐	☐	☐	☐
How often do you form new relationships with fellow on-line users?	☐	☐	☐	☐	☐
How often do others in your life complain to you about the amount of time you spend on-line?	☐	☐	☐	☐	☐
How often do your grades or school work suffers because of the amount of time you spend on-line?	☐	☐	☐	☐	☐
How often do you check your email before something else that you need to do?	☐	☐	☐	☐	☐
How often does your job performance or productivity suffer because of the Internet?	☐	☐	☐	☐	☐
How often do you become defensive or secretive when anyone asks you what you do on-line?	☐	☐	☐	☐	☐
How often do you block out disturbing thoughts about your life with soothing thoughts of the Internet?	☐	☐	☐	☐	☐
How often do you find yourself anticipating when you will go on-line again?	☐	☐	☐	☐	☐
How often do you fear that life without the Internet would be boring, empty, and joyless?	☐	☐	☐	☐	☐
How often do you snap, yell, or act annoyed if someone bothers you while you are on-line?	☐	☐	☐	☐	☐
How often do you lose sleep due to late-night log-ins?	☐	☐	☐	☐	☐
How often do you feel preoccupied with the Internet when off-line, or fantasize about being on-line?	☐	☐	☐	☐	☐
How often do you find yourself saying "just a few more minutes" when online?	☐	☐	☐	☐	☐
How often do you try to cut down the amount of time you spend on-line 1 2 3 4 5 0and fail?	☐	☐	☐	☐	☐
How often do you try to hide how long you've been on-line?	☐	☐	☐	☐	☐
How often do you choose to spend more time on-line over going out with others?	☐	☐	☐	☐	☐
How often do you feel depressed, moody or nervous when you are off-line, which goes away once you are back on-line?	☐	☐	☐	☐	☐

PARENTAL BONDING INSTRUMENT

This questionnaire lists various attitudes and behaviours of mother (natural mother/step mother/adoptive mother) and father (natural father/step father/adoptive father).

Note:

- *Do BOTH sections: MOTHER & FATHER if you live with both your father (natural father/step father/adopted father) and your mother (natural mother/stepmother/adoptive mother)*
- *Do the FATHER section if you are only living with your father (natural father/step father/adoptive father).*
- *Do the MOTHER section if you only live with your mother (natural mother/step mother/adoptive mother).*
- *Please go to the next section to answer the questions if you do not live with your father (natural father/step father/adoptive father) and mother (natural mother/step mother/adoptive mother).*

FATHER				STATEMENT	MOTHER			
Very like	Moderately like	Moderately unlike	Very unlike		Very unlike	Moderately unlike	Moderately like	Very like
☐	☐	☐	☐	Spoke to me in a warm and friendly voice	☐	☐	☐	☐
☐	☐	☐	☐	Did not help me as much as I needed	☐	☐	☐	☐
☐	☐	☐	☐	Let me do those things I liked doing	☐	☐	☐	☐
☐	☐	☐	☐	Seemed emotionally cold to me	☐	☐	☐	☐

☐	☐	☐	☐	Appeared to understand my problems and worries	☐	☐	☐	☐
☐	☐	☐	☐	Was affectionate to me	☐	☐	☐	☐
☐	☐	☐	☐	Liked me to make my own decisions	☐	☐	☐	☐
☐	☐	☐	☐	Did not want me to grow up	☐	☐	☐	☐
☐	☐	☐	☐	Tried to control everything I did	☐	☐	☐	☐
☐	☐	☐	☐	Invaded my privacy	☐	☐	☐	☐
☐	☐	☐	☐	Enjoyed talking things over with me	☐	☐	☐	☐
☐	☐	☐	☐	Frequently smiled at me	☐	☐	☐	☐
☐	☐	☐	☐	Tended to baby me	☐	☐	☐	☐
☐	☐	☐	☐	Did not seem to understand what I needed or wanted	☐	☐	☐	☐
☐	☐	☐	☐	Let me decide things for myself	☐	☐	☐	☐
☐	☐	☐		Made me feel I wasn’t wanted	☐	☐	☐	☐
☐	☐	☐	☐	Could make me feel better when I was upset	☐	☐	☐	☐
☐	☐	☐	☐	Did not talk with me very much	☐	☐	☐	☐
☐	☐	☐	☐	Tried to make me feel dependent on her/him	☐	☐	☐	☐
☐	☐	☐	☐	Felt I could not look after myself unless she/he was around	☐	☐	☐	☐
☐	☐	☐	☐	Gave me as much freedom as I wanted	☐	☐	☐	☐
☐	☐	☐	☐	Let me go out as often as I wanted	☐	☐	☐	☐
☐	☐	☐	☐	Was overprotective of me	☐	☐	☐	☐
☐	☐	☐	☐	Did not praise me	☐	☐	☐	☐
☐	☐	☐	☐	Let me dress in any way I pleased	☐	☐	☐	☐

SCHOOL CONNECTEDNESS SCALE

	**Strongly disagree**	**Disagree**	**Neutral**	**Agree**	**Strong agreely**
I feel safe in my school	☐	☐	☐	☐	☐
I feel safe in my school	☐	☐	☐	☐	☐
I am happy to be at this school	☐	☐	☐	☐	☐
I feel like I am part of this school	☐	☐	☐	☐	☐
I feel close to people at this school	☐	☐	☐	☐	☐

NEIGHBORHOOD ENVIRONMENT

How often do you experience fights, quarrel in your neighborhood?	☐ Never<br>☐ Sometimes (1-3 times/month)<br>☐ Often (≥1 time/week)	
How often do you experience crime in your neighborhood?	☐ Never<br>☐ Sometimes (1-3 times/month)<br>☐ Often (≥1 time/week)	
Which of the following behaviors do your peers have towards other people in your neighborhood? **(You can select multiple answers)**	☐ Teasing makes others uncomfortable<br>☐ Fighting<br>☐ Swearing, slandering<br>☐ Speaking out the secrets of others	☐ Threatening or forcing others to do something they don't want to do<br>☐ Breaking friendships with others or forcing others to leave the group<br>☐ None/Don't know
What is the economic status of your neighborhood?	☐ Poor<br>☐ Average<br>☐ Rich	

DEPRESSION

**DURING THE PAST WEEK**	**Rarely or none of the time (less than 1 day)**	**Some or a little of the time (1-2 days)**	**Occasionally or a moderate amount of time (3-4 days)**	**Most or all of the time (5-7 days)**

I was bothered by things that usually don't bother me	☐	☐	☐	☐
I did not feel like eating; my appetite was poor	☐	☐	☐	☐
I felt that I could not shake off the blues even with help from my family or friends	☐	☐	☐	☐
I felt I was just as good as other people	☐	☐	☐	☐
I had trouble keeping my mind on what I was doing	☐	☐	☐	☐
I felt depressed	☐	☐	☐	☐
I felt that everything I did was an effort	☐	☐	☐	☐
I felt hopeful about the future	☐	☐	☐	☐
I thought my life had been a failure	☐	☐	☐	☐
I felt fearful	☐	☐	☐	☐
My sleep was restless	☐	☐	☐	☐
I was happy	☐	☐	☐	☐
I talked less than usual	☐	☐	☐	☐
I felt lonely	☐	☐	☐	☐
People were unfriendly	☐	☐	☐	☐
I enjoyed life	☐	☐	☐	☐
I had crying spells	☐	☐	☐	☐
I felt sad	☐	☐	☐	☐
I felt that people dislike me	☐	☐	☐	☐
I could not get "going"	☐	☐	☐	☐

CYBERBULLYING SCALE

The following questions ask about your life in the **PAST FEW MONTHS**.

Cyberbullying: *Any type of harassment or bullying (teasing, telling lies, making fun of someone, making rude or mean comments, spreading rumors, or making threatening or aggressive comments) that occurs through e-mail, a chat room, instant messaging, a website (including blogs), text messaging, or videos or pictures posted on websites or sent through cell phones.*

Do other kids use any of the following to bully you?<br>**(Select Multiple Answers)**	☐ Email<br>☐ Online video clips of you<br>☐ Text messages/Twitter<br>☐ Picture messages<br>☐ Instant messaging<br>☐ Chatroom	☐ Social networking site (such as Facebook)<br>☐ Virtual world (such as Second Life or the Sims)<br>☐ Developed a mean website or message board about you
Do you use any of the following to bully other kids?<br>**(Select Multiple Answers)**	☐ Email<br>☐ Online video clips of you<br>☐ Text messages/Twitter<br>☐ Picture messages<br>☐ Instant messaging<br>☐ Chatroom	☐ Social networking site (such as Facebook)<br>☐ Virtual world (such as Second Life or the Sims)<br>☐ Developed a mean website or message board about you

PAST FEW MONTHS	Never	Almost Never	Sometimes	Almost all the time	All the time
How often do you get online or text messages from another kid threatening to beat you up or hurt you physically?	☐	☐	☐	☐	☐

How often do other kids leave you out of online groups on purpose?	☐	☐	☐	☐	☐
How often does another kid say something mean to you (such as calling you names or making fun of you) in a text message or online?	☐	☐	☐	☐	☐
How often does a kid who is mad at you try to get back at you by not letting you be in their online group anymore?	☐	☐	☐	☐	☐
How often do you get text or online messages that make you afraid for your safety?	☐	☐	☐	☐	☐
How often does a kid tell lies about you in texts or online to make other kids not like you anymore?	☐	☐	☐	☐	☐
How often does another kid say online that they won't like you unless you do what they want you to do?	☐	☐	☐	☐	☐
How often does a kid try to keep others from liking you by texting or posting mean things about you?	☐	☐	☐	☐	☐
How often does another kid send you a message saying they will beat you up if you don't do what they want you to do?	☐	☐	☐	☐	☐
How often do you get in online fights?	☐	☐	☐	☐	☐
How often does another kid put you down online by sending or posting cruel gossip, rumors, or something else hurtful?	☐	☐	☐	☐	☐
How often does another kid pretended to be you and send or post something that damages your reputation or friendships?	☐	☐	☐	☐	☐
How often does another kid share your personal secrets or images online without your permission?	☐	☐	☐	☐	☐
How often have you had to ask an adult to help fix something bad that happened to you online (like a mean picture of you was posted, people called you names, someone threatened you)?	☐	☐	☐	☐	☐

THANK YOU FOR PARTICIPATING IN THIS RESEARCH!

Vietnamese version

Những câu hỏi sau đây sẽ hỏi về các mối quan hệ trường lớp, cuộc sống gia đình, xã hội, những trải nghiệm liên quan đến bắt nạt trực tuyến và sức khỏe của chính bạn. Đây không phải là bài kiểm tra ở trường nên sẽ không có câu trả lời đúng sai. ĐỪNG CHO BẤT KỲ AI BIẾT ĐƯỢC NHỮNG CÂU TRẢ LỜI NÀY. CHÚNG TÔI SẼ GIỮ BÍ MẬT CHO NHỮNG CÂU TRẢ LỜI CỦA BẠN VÀ SẼ KHÔNG AI NHẬN RA BẠN QUA NHỮNG CÂU TRẢ LỜI. Hãy đọc những câu hỏi dưới đây và đánh dấu chọn vào câu trả lời phù hợp nhất với bạn.

THÔNG TIN BẢN THÂN

Giới tính	☐ Nam ☐ Nữ
Bạn hiện đang học lớp mấy?	☐ Lớp 8 ☐ Lớp 9 ☐ Lớp 10 ☐ Lớp 11 ☐ Lớp 12
Xếp loại học lực học kì gần nhất của bạn	☐ Kém ☐ Yếu ☐ Trung bình ☐ Khá ☐ Giỏi
Mức độ thường xuyên truy cập Internet của bạn?	☐ Hàng ngày<br>☐ Một vài lần/tuần ☐ Rất hiếm khi<br>☐ Một vài lần/tháng ☐ Không bao giờ
Thời gian truy cập Internet trung bình MỖI NGÀY của bạn khoảng bao nhiêu?	☐ <2 giờ/ngày ☐ 2-4 giờ/ngày ☐ >4 giờ/ngày
Địa điểm mà bạn thường truy cập Internet?<br><br>**(Có thể chọn nhiều câu trả lời)**	☐ Ở nhà trong phòng riêng<br>☐ Ở nhà trong phòng khách khi không có ai<br>☐ Ở nhà trong phòng khách khi có người thân<br>☐ Nơi công cộng (quán net, …)<br>☐ Ở trường trong giờ học<br>☐ Ở trường ngoài giờ học<br>☐ Nơi khác (Ghi rõ): ……………………….
Phương tiện mà bạn thường sử dụng nhất để truy cập Internet?<br>**(Có thể chọn nhiều câu trả lời)**	☐ Điện thoại di động<br>☐ Máy tính bảng<br>☐ Máy tính dùng chung<br>☐ Máy tính cá nhân<br>☐ Khác (Ghi rõ): …………………..
Bạn thường làm gì khi truy cập Internet?<br>**(Có thể chọn nhiều câu trả lời)**	☐ Truy cập các trang mạng xã hội (Facebook, Twitter, Youtube, …)<br>☐ Nói chuyện với người khác<br>☐ Gửi thư điện tử<br>☐ Tìm kiếm thông tin<br>☐ Đọc tin tức<br>☐ Học tập<br>☐ Nghe nhạc<br>☐ Xem phim<br>☐ Mua sắm<br>☐ Chơi game<br>☐ Đăng ảnh<br>☐ Khác (Ghi rõ): …………………..
Hiện tại, bạn đang sống cùng ai?<br>**(Có thể chọn nhiều câu trả lời)**	☐ Sống cùng với cả cha và mẹ<br>☐ Chỉ sống với cha/mẹ<br>☐ Sống với họ hàng<br>☐ Ở nhà trọ một mình<br>☐ Khác (Ghi rõ): ……………………………

SỬ DỤNG INTERNET

	**Không bao giờ**	**Hiếm khi**	**Thỉnh thoảng**	**Thường xuyên**	**Luôn luôn**
Bạn có thường nhận thấy mình truy cập internet (online) lâu hơn dự kiến?	☐	☐	☐	☐	☐
Bạn có thường xao nhãng việc nhà để dành thời gian truy cập internet (online)?	☐	☐	☐	☐	☐
Bạn có thường thích những trò tiêu khiển trên internet thay vì tương tác với người thân, bạn bè?	☐	☐	☐	☐	☐
Bạn có thường tạo những mối quan hệ mới với những người trên mạng?	☐	☐	☐	☐	☐
Những người xung quanh có thường than phiền về thời gian truy cập internet (online) của bạn không?	☐	☐	☐	☐	☐

Điểm số học tập hoặc công việc của bạn bị ảnh hưởng do việc sử dụng nhiều thời gian để truy cập internet (online) không?	☐	☐	☐	☐	☐
Bạn có thường kiểm tra email trước khi làm những việc khác trong ngày không?	☐	☐	☐	☐	☐
Việc học tập hoặc năng suất làm việc của bạn có thường bị ảnh hưởng bởi internet không?	☐	☐	☐	☐	☐
Bạn có thường cảm thấy đề phòng hay muốn giữ bí mật khi người khác hỏi về những việc bạn đã làm trên internet không?	☐	☐	☐	☐	☐
Bạn có thường thay thế những suy nghĩ lo âu trong cuộc sống bằng những suy nghĩ dễ chịu hơn về việc sử dụng internet không?	☐	☐	☐	☐	☐
Bạn có thường mong đợi sẽ được tiếp tục truy cập internet?	☐	☐	☐	☐	☐
Bạn có cảm thấy nếu không có internet cuộc sống sẽ thật nhàm chán, trống rỗng và vô vị không?	☐	☐	☐	☐	☐
Bạn có thường nổi cáu, la hét hoặc cư xử thô lỗ khi người khác làm gián đoạn lúc đang truy cập internet (online) không?	☐	☐	☐	☐	☐
Bạn có thường mất ngủ vì truy cập internet (online) khuya không?	☐	☐	☐	☐	☐
Bạn có thường bận tâm tới internet khi không sử dụng hoặc tưởng tượng tới việc đang được truy cập internet (online) không?	☐	☐	☐	☐	☐
Bạn có tự nhủ "chỉ lên internet thêm vài phút nữa" không?	☐	☐	☐	☐	☐
Bạn có thường cố gắng giảm thời gian truy cập internet (online) nhưng thất bại không?	☐	☐	☐	☐	☐
Bạn có thường che giấu thời lượng truy cập internet (online) thực sự của mình không?	☐	☐	☐	☐	☐
Bạn có thường dành nhiều thời gian để truy cập internet (online) hơn là đi chơi với bạn bè không?	☐	☐	☐	☐	☐
Bạn có cảm giác buồn chán, lo lắng khi không sử dụng internet và cảm giác này sẽ mất khi bạn sử dụng trở lại?	☐	☐	☐	☐	☐

SỰ GẮN KẾT VỚI CHA MẸ

Những câu dưới đây nói về thái độ và cư xử của cha (cha ruột/cha kế/cha nuôi), mẹ (mẹ ruột/mẹ kế/mẹ nuôi) đối với bạn trong cuộc sống hàng ngày.

Lưu ý:

- *Với những bạn đang sống chung với CẢ cha (cha ruột/cha kế/cha nuôi) và mẹ (mẹ ruột/mẹ kế/mẹ nuôi) thì làm CẢ hai phần: ĐỐI VỚI CHA và ĐỐI VỚI MẸ*
- *Với những bạn CHỈ đang sống chung với cha (cha ruột/cha kế/cha nuôi) thì làm phần ĐỐI VỚI CHA*
- *Với những bạn CHỈ đang sống chung với mẹ (mẹ ruột/mẹ kế/mẹ nuôi) thì làm phần ĐỐI VỚI MẸ*
- *Với những bạn đang KHÔNG sống chung với cha (cha ruột/cha kế/cha nuôi) và mẹ (mẹ ruột/mẹ kế/mẹ nuôi) thì KHÔNG làm cả hai phần ĐỐI VỚI CHA và ĐỐI VỚI MẸ. Mời bạn chuyển sang phần kế tiếp để tiếp tục trả lời.*

ĐỐI VỚI **CHA**				**NỘI DUNG**	ĐỐI VỚI **MẸ**			
**Rất đúng**	**Gần đúng**	**Gần sai**	**Rất sai**		**Rất sai**	**Gần sai**	**Gần đúng**	**Rất đúng**
☐	☐	☐	☐	Nói chuyện với tôi một cách ấm áp, thân thiện	☐	☐	☐	☐
☐	☐	☐	☐	Không giúp đỡ tôi nhiều như tôi mong	☐	☐	☐	☐
☐	☐	☐	☐	Để tôi làm điều tôi thích	☐	☐	☐	☐
☐	☐	☐	☐	Tỏ ra lạnh nhạt với tôi	☐	☐	☐	☐
☐	☐	☐	☐	Tỏ ra hiểu những vấn đề tôi nói	☐	☐	☐	☐

☐	☐	☐	☐	Thái độ trìu mến với tôi	☐	☐	☐	☐
☐	☐	☐	☐	Để tôi tự đưa ra quyết định	☐	☐	☐	☐
☐	☐	☐	☐	Không muốn tôi trưởng thành quá nhanh (bảo bọc)	☐	☐	☐	☐
☐	☐	☐	☐	Cố gắng kiểm soát những việc tôi làm	☐	☐	☐	☐
☐	☐	☐	☐	Không cho tôi sự riêng tư và bí mật	☐	☐	☐	☐
☐	☐	☐	☐	Thường hay chỉ bảo tôi quá nhiều	☐	☐	☐	☐
☐	☐	☐	☐	Hay mỉm cười với tôi	☐	☐	☐	☐
☐	☐	☐	☐	Hay xem tôi như trẻ con	☐	☐	☐	☐
☐	☐	☐	☐	Không hiểu những gì tôi cần và tôi muốn	☐	☐	☐	☐
☐	☐	☐	☐	Để tôi quyết định những việc của bản thân	☐	☐	☐	☐
☐	☐	☐	☐	Khiến tôi cảm thấy không ai cần đến tôi	☐	☐	☐	☐
☐	☐	☐	☐	Làm tôi cảm thấy tốt hơn khi tôi đang khó chịu	☐	☐	☐	☐
☐	☐	☐	☐	Ít khi nói chuyện với tôi	☐	☐	☐	☐
☐	☐	☐	☐	Khiến tôi cảm thấy bị phụ thuộc nhiều	☐	☐	☐	☐
☐	☐	☐	☐	Khiến tôi cảm thấy không thể tự chăm sóc mình	☐	☐	☐	☐
☐	☐	☐	☐	Cho tôi nhiều tự do	☐	☐	☐	☐
☐	☐	☐	☐	Cho tôi đi ra ngoài đường thường xuyên như tôi muốn	☐	☐	☐	☐
☐	☐	☐	☐	Thường tỏ ra quá bảo vệ tôi	☐	☐	☐	☐
☐	☐	☐	☐	Ít khen ngợi tôi	☐	☐	☐	☐
☐	☐	☐	☐	Để tôi mặc theo cách tôi thích	☐	☐	☐	☐

SỰ GẮN KẾT VỚI TRƯỜNG LỚP

	**Hoàn toàn không đồng ý**	**Không đồng ý**	**Không biết**	**Đồng ý**	**Hoàn toàn đồng ý**
Tôi cảm thấy an toàn ở trường tôi	☐	☐	☐	☐	☐
Giáo viên trong trường đối xử công bằng với học sinh	☐	☐	☐	☐	☐
Tôi thấy vui khi học tại trường này	☐	☐	☐	☐	☐
Tôi rất thích là học sinh của trường này	☐	☐	☐	☐	☐
Tôi cảm thấy gần gũi với mọi người trong trường	☐	☐	☐	☐	☐

MÔI TRƯỜNG SỐNG VÀ TRẢI NGHIỆM BẠO LỰC

Khu vực bạn đang sống có xảy ra đánh nhau, cãi nhau không?	☐ Chưa bao giờ<br>☐ Thỉnh thoảng (1-3 lần/tháng)<br>☐ Thường xuyên (ít nhất 1 lần/tuần)
Khu vực bạn đang sống có xảy ra các tệ nạn như trộm cắp, cướp của, cờ bạc…	☐ Chưa bao giờ<br>☐ Thỉnh thoảng (1-3 lần/tháng)<br>☐ Thường xuyên (ít nhất 1 lần/tuần)
Những người bạn trong xóm/cùng nhà trọ của bạn có hành vi nào sau đây đối với những người khác?<br>**(Có thể chọn nhiều câu trả lời)**	☐ Trêu chọc làm người khác khó chịu<br>☐ Đánh nhau<br>☐ Chửi mắng, nói xấu.<br>☐ Nói ra bí mật của người khác<br>☐ Đe dọa hoặc bắt người khác làm điều họ không muốn.<br>☐ Phá vỡ tình bạn của người khác hoặc buộc người khác rời khỏi nhóm.<br>☐ Không có/Không biết
Hầu hết hàng xóm khu vực nhà bạn có đời sống kinh tế như thế nào?	☐ Nghèo<br>☐ Trung bình – Khá

	☐ Giàu

TRẦM CẢM

TRONG TUẦN VỪA QUA	Hiếm khi (<1 ngày)	Một vài lần (1-2 ngày)	Thỉnh thoảng (3-4 ngày)	Hầu hết thời gian (5-7 ngày)
Tôi dễ bực bội vì những điều thường ngày không gây bực cho tôi	☐	☐	☐	☐
Tôi cảm thấy không muốn ăn và ăn không thấy ngon	☐	☐	☐	☐
Tôi cảm thấy không thể quên nỗi buồn mặc dù gia đình, bạn bè giúp đỡ tôi	☐	☐	☐	☐
Tôi cảm thấy tôi là người tốt (khỏe mạnh) như những người khác	☐	☐	☐	☐
Tôi hay quên những việc tôi đang làm	☐	☐	☐	☐
Tôi cảm thấy bị trầm cảm	☐	☐	☐	☐
Tôi thấy mọi việc tôi làm là sai	☐	☐	☐	☐
Tôi cảm thấy tràn đầy hi vọng về tương lai	☐	☐	☐	☐
Tôi cảm thấy cuộc đời tôi đã từng thất bại	☐	☐	☐	☐
Tôi thấy sợ hãi	☐	☐	☐	☐
Tôi ngủ không yên giấc	☐	☐	☐	☐
Tôi vui vẻ	☐	☐	☐	☐
Tôi nói chuyện ít hơn bình thường	☐	☐	☐	☐
Tôi cảm thấy cô đơn	☐	☐	☐	☐
Mọi người không thân thiện với tôi	☐	☐	☐	☐
Tôi yêu thích cuộc sống hiện tại	☐	☐	☐	☐
Tôi khóc nhiều lần	☐	☐	☐	☐
Tôi thấy buồn	☐	☐	☐	☐
Tôi cảm thấy mọi người ghét tôi	☐	☐	☐	☐
Tôi không thể tiếp tục mọi việc như bình thường được nữa	☐	☐	☐	☐

BẮT NẠT TRỰC TUYẾN

Những câu dưới đây hỏi về tình trạng bị bắt nạt trực tuyến trong cuộc sống của bạn ***MỘT VÀI THÁNG QUA****.*
Bị bắt nạt trực tuyến: là bất kỳ loại quấy rối nào như trêu ghẹo, bịa chuyện, châm chọc, đưa ra bình luận thô lỗ, xúc phạm, truyền bá tin đồn, hoặc đưa ra các lời đe dọa, cảnh cáo xảy ra thông qua email, phòng chat, nhắn tin qua mạng, trang web (bao gồm cả blog) hoặc tin nhắn điện thoại.

Những bạn khác có sử dụng bất kỳ hình thức nào dưới đây để bắt nạt bạn hay không?<br>**(Có thể chọn nhiều câu trả lời)**	☐ Email<br>☐ Đăng phim ghi hình của bạn<br>☐ Tin nhắn điện thoại/Twitter<br>☐ Trang mạng xã hội (Facebook, …)<br>☐ Tin nhắn hình ảnh<br>☐ Nhóm chat<br>☐ Nhắn tin qua mạng<br>☐ Trò chơi thực tế ảo (ví dụ như Second Life hoặc The Sims)<br>☐ Tạo ra một trang web hoặc diễn đàn trực tuyến nói xấu bạn<br>☐ Không có
Bạn có sử dụng bất kỳ hình thức nào dưới đây để bắt nạt các bạn khác hay không?<br>**(Có thể chọn nhiều câu trả lời)**	☐ Email<br>☐ Đăng phim ghi hình của bạn<br>☐ Tin nhắn điện thoại/Twitter<br>☐ Trang mạng xã hội (Facebook, …)<br>☐ Tin nhắn hình ảnh

	☐ Nhóm chat<br>☐ Nhắn tin qua mạng<br>☐ Trò chơi thực tế ảo (ví dụ như Second Life hoặc The Sims)<br>☐ Tạo ra một trang web hoặc diễn đàn trực tuyến nói xấu bạn<br>☐ Không có

**TRONG VÀI THÁNG QUA**	**Không bao giờ**	**Gần như không**	**Thỉnh thoảng**	**Gần như thường xuyên**	**Luôn luôn**
Bạn có thường nhận được tin nhắn điện thoại/qua mạng từ bạn khác có nội dung dọa đánh hay làm tổn thương bạn về thể chất không?	☐	☐	☐	☐	☐
Những bạn khác có thường cố ý tẩy chay bạn trong các nhóm/cộng đồng trên mạng không?	☐	☐	☐	☐	☐
Những bạn khác có thường nói những điều không tốt về bạn (ví dụ như đặt biệt danh hoặc đem bạn ra làm trò đùa) bằng tin nhắn điện thoại/qua mạng không?	☐	☐	☐	☐	☐
Nếu một người bạn khác tức giận với bạn, người đó có trả đũa bạn bằng cách không để bạn tham gia nhóm bạn bè trên mạng của họ nữa hay không?	☐	☐	☐	☐	☐
Bạn có thường nhận được tin nhắn điện thoại/qua mạng khiến bạn lo lắng về sự an toàn của bạn không?	☐	☐	☐	☐	☐
Bạn có thường bị một người bạn khác dùng tin nhắn điện thoại/qua mạng để nói sai sự thật về bạn làm cho những người bạn khác không còn thích bạn nữa không?	☐	☐	☐	☐	☐
Bạn có thường bị một người bạn khác nói trên mạng là họ sẽ không thích bạn trừ khi bạn làm theo những gì họ muốn không?	☐	☐	☐	☐	☐
Bạn có thường bị một người bạn khác sử dụng tin nhắn hoặc bài đăng để nói xấu về bạn và khiến những người khác không thích bạn không?	☐	☐	☐	☐	☐
Bạn có thường bị một người bạn khác gửi tin nhắn với nội dung dọa đánh nếu như bạn không làm theo những gì họ muốn không?	☐	☐	☐	☐	☐
Bạn có thường bị vướng vào xung đột trên mạng không?	☐	☐	☐	☐	☐
Những người bạn khác có thường làm bạn xấu hổ trên mạng bằng cách gửi hoặc đăng những lời bàn tán, tin đồn cay nghiệt, hoặc những thông tin khác khiến bạn bị tổn thương không?	☐	☐	☐	☐	☐
Những người bạn khác có thường giả mạo bạn để gửi hoặc đăng thông tin gây tổn hại đến danh dự hoặc các mối quan hệ bạn bè của bạn không?	☐	☐	☐	☐	☐
Những người bạn khác có thường chia sẻ bí mật hoặc hình ảnh cá nhân của bạn lên mạng mà không có sự đồng ý của bạn không?	☐	☐	☐	☐	☐
Bạn có thường phải nhờ người lớn giải quyết những tình huống xấu xảy ra với bạn trên mạng không (ví dụ như một bức ảnh không tốt của bạn bị đăng lên mạng, mọi người gọi bạn bằng biệt danh, có ai đó đe dọa bạn?)	☐	☐	☐	☐	☐

XIN CHÂN THÀNH CẢM ƠN BẠN ĐÃ THAM GIA NGHIÊN CỨU!
