# Supplementary material for: Cyber-victimization and its association with depression among Vietnamese adolescents
Source: PeerJ. 2022 Feb 9;10:e12907. doi: 10.7717/peerj.12907 (PMC8840053; doi:10.7717/peerj.12907)
Supplement: Supplemental Information 3 [file peerj-10-12907-s003.docx]

Table 1A: Characteristics and Internet Use Behaviors Among Vietnamese Adolescents, Stratified by Symptoms of Depression and Sex.

| **Characteristics** | **MALE** | |  | **FEMALE** | |
| --- | --- | --- | --- | --- | --- |
|  | **Symptoms of depression** | |  | **Symptoms of depression** | |
|  | ***Yes*** | ***No*** |  | ***Yes*** | ***No*** |
|  | *n (Weighted %, 95% CI)* | *n (Weighted %, 95% CI)* |  | *n (Weighted %, 95% CI)* | *n (Weighted %, 95% CI)* |
| **Background information** | | | | | |
| **Grade** | *χ^2^ (df=1)=22.5; F(1, 39)=29.0; p<0.001* | |  | *χ^2^ (df=1)=11.5; F(1, 39)=11.1; p=0.002* | |
| High school | 178 (77.5, 62.2 - 87.8) | 193 (60.2, 42.4 - 75.7) |  | 282 (80.2, 66.3 - 89.3) | 239 (70.0, 53.5 - 82.5) |
| Secondary school | 90 (22.5, 12.2 - 37.8) | 216 (39.8, 24.3 - 57.6) |  | 123 (19.8, 10.7 - 33.7) | 171 (30.0, 17.5 - 46.5) |
| **Grade point average in the last semester** | *χ^2^ (df=2)=0.5; F(1.9, 72.5)=0.2; p=0.803* | |  | *χ^2^ (df=2)=7.0; F(2.0, 75.9)=4.2; p=0.020* | |
| Very good/Excellent (≥8/10) | 61 (23.5, 17.8 - 30.4) | 112 (25.8, 19.7 - 33.0) |  | 157 (37.8, 28.4 - 48.2) | 187 (44.6, 34.3 - 55.3) |
| Average/Good (≥7/10-8/10) | 142 (54.0, 47.8 - 60.1) | 202 (52.6, 45.3 - 59.7) |  | 169 (44.5, 37.0 - 52.2) | 170 (43.6, 35.2 - 52.4) |
| Low (<7/10) | 65 (22.5, 16.4 - 30.0) | 95 (21.6, 15.5 - 29.4) |  | 79 (17.7, 12.8 - 23.9) | 53 (11.8, 8.2 - 16.8) |
| **Internet use behaviors** | | | | | |
| **Using internet every day** | *χ^2^ (df=1)=3.2; F(1, 39)=3.0; p=0.091* | |  | *χ^2^ (df=1)=1.3; F(1, 39)=0.7; p=0.410* | |
| Yes | 235 (88.0, 83.1 - 91.6) | 370 (92.1, 87.7 - 95.0) |  | 374 (93.2, 89.0 - 95.9) | 387 (95.1, 91.9 - 97.1) |
| No | 33 (12.0, 8.4 - 16.9) | 39 (7.9, 5.0 - 12.3) |  | 31 (6.8, 4.1 - 11.0) | 23 (4.9, 2.9 - 8.1) |
| **Time spent on using internet (hours/day)** | *χ^2^ (df=2)=3.4; F(1.8, 71.5)=1.9; p=0.165* | |  | *χ^2^ (df=2)=5.5; F(1.9, 75.6)=2.8; p=0.066* | |
| <2 | 45 (17.2, 13.2 - 22.1) | 90 (20.8, 17.4 - 24.7) |  | 56 (12.3, 8.5 - 17.5) | 56 (12.4, 9.1 - 16.7) |
| 2-4 | 124 (48.0, 42.0 - 54.0) | 197 (50.5, 45.4 - 55.7) |  | 164 (40.7, 35.0 - 46.6) | 199 (48.3, 44.0 - 52.7) |
| >4 | 99 (34.8, 30.4 - 39.5) | 122 (28.6, 24.6 - 33.0) |  | 185 (47.0, 39.7 - 54.5) | 155 (39.2, 33.4 - 45.4) |
| **Internet addiction** | *χ^2^ (df=2)=59.7; F(1.7, 67.2)=19.1; p<0.001* | |  | *χ^2^ (df=2)=74.4; F(1.9, 72.4)=38.1; p<0.001* | |
| No | 65 (24.1, 17.6 - 32.1) | 214 (51.4, 47.8 - 55.0) |  | 109 (25.9, 20.9 - 31.5) | 223 (54.3, 49.8 - 58.8) |
| Mild/moderate | 190 (71.0, 63.5 - 77.5) | 194 (48.1, 44.2 - 52.1) |  | 280 (69.8, 64.6 - 74.5) | 184 (45.1, 40.8 - 49.4) |
| Severe | 13 (4.9, 2.8 - 8.4) | 1 (0.5, 0.1 - 3.3) |  | 16 (4.3, 2.2 - 8.2) | 3 (0.6, 0.2 - 2.0) |
