# Supplementary material for: Cyber-victimization and its association with depression among Vietnamese adolescents
Source: PeerJ. 2022 Feb 9;10:e12907. doi: 10.7717/peerj.12907 (PMC8840053; doi:10.7717/peerj.12907)
Supplement: Supplemental Information 4 [file peerj-10-12907-s004.docx]

Table 2A: Living and Studying Environment and Cyber Victimization Among Vietnamese Adolescents, Stratified by Symptoms of Depression and Sex.

| **Characteristics** | **MALE** | |  | **FEMALE** | |
| --- | --- | --- | --- | --- | --- |
|  | **Symptoms of depression** | |  | **Symptoms of depression** | |
|  | ***Yes*** | ***No*** |  | ***Yes*** | ***No*** |
|  | *n (Weighted %, 95% CI)* | *n (Weighted %, 95% CI)* |  | *n (Weighted %, 95% CI)* | *n (Weighted %, 95% CI)* |
| **Family environment** | | | | | |
| **Living with whom** | *χ^2^ (df=2)=24.8; F(2.0, 76.2)=16.1; p<0.001* | |  | *χ^2^ (df=2)=8.1; F(1.8, 70.7)=2.8; p=0.070* | |
| With parents | 207 (73.6, 66.3 - 79.8) | 367 (88.3, 84.2 - 91.5) |  | 336 (81.6, 75.0 - 86.8) | 365 (88.4, 84.3 - 91.6) |
| With either mother or father | 48 (20.7, 15.2 - 27.5) | 35 (9.7, 7.0 - 13.1) |  | 54 (14.4, 9.8 - 20.8) | 37 (9.8, 7.0 - 13.6) |
| With others | 13 (5.7, 3.7 - 8.7) | 7 (2.0, 0.9 - 4.2) |  | 15 (4.0, 2.6 - 6.0) | 8 (1.8, 0.8 - 3.7) |
| **Parental bonding** |  | |  |  |  |
| Caring from father | *χ^2^ (df=1)=63.0; F(1, 39)=88.2; p<0.001* | |  | *χ^2^ (df=1)=80.8; F(1, 39)=73.7; p<0.001* | |
|  | 65 (25.5, 20.0 - 31.8) | 232 (57.8, 51.4 - 63.9) |  | 114 (30.4, 25.2 - 36.2) | 247 (63.3, 56.4 - 69.7) |
| Control from father | *χ^2^ (df=1)=30.8; F(1, 39)=19.9; p<0.001* | |  | *χ^2^ (df=1)=7.8; F(1, 39)=7.9; p=0.008* | |
|  | 185 (79.1, 72.5 - 84.5) | 221 (57.7, 51.4 - 63.8) |  | 239 (64.5, 58.3 - 70.4) | 202 (54.5, 49.3 - 59.6) |
| Caring from mother | *χ^2^ (df=1)=85.3; F(1, 39)=83.1; p<0.001* | |  | *χ^2^ (df=1)=62.2; F(1, 39)=55.8; p<0.001* | |
|  | 41 (16.4, 11.9 - 22.2) | 222 (53.1, 46.9 - 59.2) |  | 96 (26.1, 20.7 - 32.3) | 215 (53.9, 48.2 - 59.5) |
| Control from mother | *χ^2^ (df=1)=43.4; F(1, 39)=39.4; p<0.001* | |  | *χ^2^ (df=1)=33.3; F(1, 39)=28.9; p<0.001* | |
|  | 185 (80.5, 74.8 - 85.1) | 214 (54.8, 49.5 - 60.0) |  | 263 (70.8, 65.4 - 75.8) | 201 (50.5, 45.2 - 55.9) |
| **Neighborhood environment** | | | | | |
| **Economical classification of living location** | *χ^2^ (df=2)=0.9; F(2.0, 77.3)=0.4; p=0.676* | |  | *χ^2^ (df=2)=7.7; F(2.0, 77.5)=4.2; p=0.019* | |
| Poor | 11 (4.4, 2.2 - 8.7) | 14 (3.4, 2.2 - 5.3) |  | 15 (3.8, 2.2 - 6.5) | 6 (1.6, 0.7 - 3.7) |
| Average | 244 (90.9, 86.3 - 94.1) | 373 (90.8, 87.4 - 93.4) |  | 358 (88.2, 83.9 - 91.4) | 387 (93.7, 90.3 - 95.9) |
| Rich | 13 (4.7, 2.7 - 8.0) | 22 (5.8, 3.9 - 8.6) |  | 32 (8.0, 5.6 - 11.4) | 17 (4.7, 2.8 - 7.9) |
| **Often experience fight, quarrel in the neighborhood** | *χ^2^ (df=1)=3.0; F(1, 39)=1.3; p=0.255* | |  | *χ^2^ (df=1)=4.9; F(1, 39)=4.9; p=0.033* | |
| Yes | 177 (66.7, 58.2 - 74.2) | 244 (60.2, 53.2 - 66.7) |  | 278 (68.0, 62.1 - 73.3) | 249 (60.5, 55.5 - 65.4) |
| No | 91 (33.3, 25.8 - 41.8) | 165 (39.8, 33.3 - 46.8) |  | 127 (32.0, 26.7 - 37.9) | 161 (39.5, 34.6 - 44.5) |
| **Often experience crime in the neighborhood** | *χ^2^ (df=1)=1.24; F(1, 39)=0.4; p=0.524* | |  | *χ^2^ (df=1)=13.3; F(1, 39)=8.7; p=0.005* | |
| Yes | 162 (60.5, 50.2 - 70.0) | 219 (56.2, 49.0 - 63.2) |  | 262 (65.4, 57.8 - 72.3) | 212 (52.9, 47.9 - 57.8) |
| No | 106 (39.5, 30.0 - 49.8) | 190 (43.8, 36.8 - 51.0) |  | 143 (34.6, 27.7 - 42.2) | 198 (47.1, 42.2 - 52.1) |
| **Often witness violence among peers in the neighborhood** | *χ^2^ (df=1)=10.5; F(1, 39)=14.5; p<0.001* | |  | *χ^2^ (df=1)=14.7; F(1, 39)=9.5; p=0.004* | |
| Yes | 100 (38.8, 33.7 - 44.1) | 103 (27.0, 23.2 - 31.2) |  | 162 (41.5, 35.0 - 48.2) | 112 (28.6, 24.6 - 33.1) |
| No | 168 (61.2, 55.9 - 66.3) | 306 (73.0, 68.8 - 76.8) |  | 243 (58.5, 51.8 - 65.0) | 298 (71.4, 66.9 - 75.4) |
| **School environment** | | | | | |
| **School connectedness score** *(Mean & 95% CI)* | *F(1, 39)=19.5; p<0.001* | |  | *F(1, 39)=63.4; p<0.001* | |
|  | 18.0 (17.4 - 18.5) | 19.8 (19.2 - 20.4) |  | 17.8 (17.3 - 18.3) | 19.8 (19.4 - 20.3) |
| **Cyber victimization** | | | | | |
| **Cyber victimization** | *χ^2^ (df=1)=38.5; F(1, 39)=30.4; p<0.001* | |  | *χ^2^ (df=1)=44.2; F(1, 39)=30.4; p<0.001* | |
| Yes | 127 (47.7, 41.6 - 53.9) | 101 (24.8, 20.2 - 30.0) |  | 194 (49.0, 41.1 - 57.0) | 106 (26.4, 21.2 - 32.3) |
| No | 141 (52.3, 46.1 - 58.4) | 308 (75.2, 70.0 - 79.8) |  | 211 (51.0, 43.0 - 58.9) | 304 (73.6, 67.7 - 78.8) |
| **Number of cyber-victimized forms experienced** | *χ^2^ (df=4)=41.6; F(3.1, 121.8)=9.5; p<0.001* | |  | *χ^2^ (df=4)=51.5; F(3.1, 135.5)=11.8; p<0.001* | |
| 0 | 141 (52.3, 46.1 - 58.4) | 308 (75.2, 70.0 - 79.8) |  | 211 (51.0, 43.0 - 58.9) | 304 (73.6, 67.7 - 78.8) |
| 1 | 48 (16.0, 12.4 - 20.3) | 45 (10.7, 8.1 - 14.2) |  | 66 (14.8, 11.4 - 19.1) | 42 (10.3, 7.7 - 13.6) |
| 2 | 35 (14.2, 10.7 - 18.7) | 24 (5.7, 3.7 - 8.7) |  | 43 (11.2, 7.8 - 15.9) | 34 (8.0, 5.7 - 11.3) |
| 3 | 19 (7.4, 5.3 - 10.2) | 16 (4.2, 2.8 - 6.3) |  | 49 (12.2, 9.1 - 16.3) | 15 (3.6, 2.0 - 6.4) |
| 4+ | 25 (10.1, 6.8 - 14.9) | 16 (4.1, 2.3 - 7.3) |  | 36 (10.7, 7.3 - 15.4) | 15 (4.4, 2.6 - 7.5) |
