# Supplementary material for: Cyber-victimization and its association with depression among Vietnamese adolescents
Source: PeerJ. 2022 Feb 9;10:e12907. doi: 10.7717/peerj.12907 (PMC8840053; doi:10.7717/peerj.12907)
Supplement: Supplemental Information 5 [file peerj-10-12907-s005.docx]

**Data codebook**

| **Variable name** | **Variable values** | **Variable label** |
| --- | --- | --- |
| **gioi** | 0=Male; 1=Female; | Sex |
| **khoilop** | 0=Secondary school; 1=High school; | Grade |
| **hocluc** | 1=Good/Excellent (≥8/10); 2=Average (≥7/10-<8/10); 3=Low (<7/10); | Grade point average |
| **mucdo_sd** | 0=No; 1=Yes; | Using internet everyday |
| **thoigian_sd** | 1=<2; 2=2-4; 3=>4; | Time spend on using internet (hour/day) |
| **nha_phongrieng** | 0=No; 1=Yes; | Private room |
| **nha_phongkhach_khongai** | 0=No; 1=Yes; | Living room alone |
| **nha_phongkhach_conguoi** | 0=No; 1=Yes; | Living room with others |
| **noicongcong** | 0=No; 1=Yes; | Public places |
| **tronggiohoc** | 0=No; 1=Yes; | In the classroom |
| **ngoaigiohoc** | 0=No; 1=Yes; | Outside the classroom |
| **diadiem_khac** | 0=No; 1=Yes; | Others |
| **dtdd** | 0=No; 1=Yes; | Mobile phone |
| **ipad** | 0=No; 1=Yes; | Tablet |
| **maytinh_chung** | 0=No; 1=Yes; | Public computer |
| **maytinh_canhan** | 0=No; 1=Yes; | Private computer |
| **phuongtien_khac** | 0=No; 1=Yes; | Others |
| **mxh** | 0=No; 1=Yes; | Access social networks |
| **noichuyen** | 0=No; 1=Yes; | Talk with other people |
| **guimail** | 0=No; 1=Yes; | Send email |
| **timtin** | 0=No; 1=Yes; | Search for information |
| **doctin** | 0=No; 1=Yes; | Read news |
| **hoctap** | 0=No; 1=Yes; | Study |
| **nghenhac** | 0=No; 1=Yes; | Listen to music |
| **xemphim** | 0=No; 1=Yes; | Watch movies |
| **muasam** | 0=No; 1=Yes; | Shopping |
| **choigame** | 0=No; 1=Yes; | Play game |
| **danganh** | 0=No; 1=Yes; | Post photos |
| **mucdich_khac** | 0=No; 1=Yes; | Others |
| **SCS1** | 0=No; 1=Yes; | Feel safe in my school |
| **SCS2** | 0=No; 1=Yes; | The teachers at this school treat students fairly |
| **SCS3** | 0=No; 1=Yes; | Be happy to be at this school |
| **SCS4** | 0=No; 1=Yes; | Feel like I am part of this school |
| **SCS5** | 0=No; 1=Yes; | Feel close to people at this school |
| **kvs_danhnhau** | 0=No; 1=Yes; | Often experience fight, quarrel in the neighborhood |
| **kvs_tenan** | 0=No; 1=Yes; | Often experience crime in neighborhood |
| **kvs_kinhte** | 0=Poor; 1=Average; 2=Rich; | Economical classification of living location |
| **quan** | 0=Sub-urban; 1=Urban; | Living location |
| **quantamcha** | 0=No; 1=Yes; | Caring from father |
| **baovecha** | 0=No; 1=Yes; | Control from father |
| **quantamme** | 0=No; 1=Yes; | Caring from mother |
| **baoveme** | 0=No; 1=Yes; | Control from mother |
| **bancungxom** | 0=No; 1=Yes; | Bullying in the neighbour |
| **bibntt** | 0=No; 1=Yes; | Cyber victimization |
| **sl_hth_bibntt** | 0=0; 1=1; 2=2; 3=3; 4=4+; | Number of forms being vyber-victimized |
| **tramcam** | 0=No; 1=Yes; | Symptoms of depression |
| **lop** | Text | Class |
| **weight** | Numeric (N = 1492; from: 3.26 to 14.33) | Sampling weight |
| **songchung** | 1=With parents; 2=With either mother or father; 3=With others; | Living with whom |
| **int_addict** | 0=No; 1=Yes; | Internet addiction |
| **int_addict_gp** | 0=No; 1=Mild/moderate; 2=Severe; | Internet addiction |
| **vio_peer** | 0=No; 1=Yes; | Experience violence from peers in the neighborhood |
| **school_connect** | Numeric (N = 1492; from: 5 to 25) | School connectedness score |
